# Development and Validation of an Observational Behavior Tool (LIFE^™^) for Assessing Canine Coping in Shelter Environments

**DOI:** 10.3390/ani16142182

**Published:** 2026-07-14

**Authors:** Meghan E. Herron, Allison Martin, Seana Dowling-Guyer

**Affiliations:** 1 Gigi’s, Canal Winchester, OH 43110, USA; allisonmartin.msc@gmail.com; 2 Center for Animals and Public Policy, Cummings School of Veterinary Medicine at Tufts University, Medford, MA 02155, USA; seana.dowling_guyer@tufts.edu

**Keywords:** behavior, welfare, dog, inter-rater reliability, validity, dog–human interactions, dog–dog interactions

## Abstract

As animal shelters strive to promote positive welfare and successfully place dogs into adoptive homes as functional pets, the ability to identify dogs that may not be coping well within the shelter is critical. Although traditional behavior evaluations may serve multiple purposes, one of their primary purposes in animal shelters has been to predict how dogs may behave after adoption. Most traditional tests utilize a series of provocative and often artificial interactions, despite mixed evidence to support their predictive value. This study examined the reliability and validity of the LIFE (Living Interactions and Functionality Evaluation) tool, an observational assessment designed to evaluate canine coping abilities in real time within a shelter setting and identify dogs in need of intervention. Over 800 dogs were evaluated during routine shelter interactions, including kennel handling, leash walking, and interactions with people and other dogs. Observable behaviors associated with positive or negative emotional states were assigned numerical values to generate objective coping scores, which were then compared with subjective classifications assigned by trained evaluators and an expert in animal behavior. Objective scores increased significantly across subjective classification categories and aligned closely with expert assessments across all evaluated contexts. Inter-rater reliability was high, supporting consistency between evaluators. These findings support the LIFE tool as a valid and reliable welfare-focused assessment approach for identifying dogs experiencing coping difficulties within shelter environments and guiding more targeted intervention strategies, with or without a behavior expert on staff.

## Simple Summary

Formal behavior tests in animal shelters have been used for a variety of purposes, including helping to predict how dogs may behave after adoption. However, many of these tests involve stressful or artificial situations that may not accurately reflect a dog’s normal behavior. This study evaluated the LIFE (Living Interactions and Functionality Evaluation) tool, an observational assessment designed to measure how well dogs are coping within the shelter environment at a given point in time. The tool evaluates dogs during routine shelter activities such as kennel handling, leash walking, human interaction, and interaction with other dogs. Numerical scores based on observed behaviors were compared with assessments made by trained staff members and an animal behavior expert. Results showed strong agreement between scores and expert evaluations, as well as high consistency between different evaluators. The findings suggest that the LIFE tool provides a practical and objective way to assess coping in dogs and identify dogs in need. Importantly, the tool is not intended to predict adoptability or future behavior in the home, but instead to support early intervention and improve welfare outcomes for sheltered dogs.

## 1. Introduction

Shelters have long relied on standardized behavior assessments for a variety of purposes, including determining handling and personnel needs, informing adoption and length-of-stay indicators, and identifying dogs requiring behavioral intervention [1,2,3]. Despite these diverse applications, research on the most widely accepted assessments has focused predominantly on their ability to predict post-adoption behavior [2,3,4,5]. While some traditional shelter behavior evaluations demonstrate predictive value for behaviors associated with fear and anxiety, their predictive validity for more complex behaviors, particularly aggression, as well as reliability, has historically been poor [6,7]. Equally concerning, the majority of standardized assessments depend on provocative and artificial interactions [3,5,8,9] that may not reflect naturally occurring situations encountered by dogs in either the shelter or adoptive home, raising questions about both their ecological validity and their impact on animal welfare. Early work by Christensen et al. (2007) reported a reasonable negative predictive value for owner-directed aggression and resource guarding, meaning dogs who did not demonstrate aggression during a formalized in-shelter test showed low levels of aggression in the adoptive home in these two circumstances [10]. This study used a formalized testing protocol [Assess-A-Pet™ (Tawser Dog, LLC, Boise, ID)] with dogs exhibiting aggression excluded from adoption, making a positive finding of aggression difficult to validate. This same study, though, found that more than half of dogs deemed suitable for adoption were later reported to display stranger-directed aggression. Subsequent research in shelters that adopted out dogs exhibiting resource guarding behaviors found that adopters frequently did not perceive post-adoption resource guarding as problematic [11], and one shelter observed a decrease in return rates after removing resource guarding from formal evaluations [12]. A review of published behavior assessment outcomes regarding sheltered dogs concluded that, mathematically, aggression identified during standardized behavior tests would predict post-adoption aggression only slightly better than chance accuracy [13]. Collectively, these findings call into question the validity of formal behavior evaluations as predictors of certain in-home behavior and suggest that greater emphasis should be placed on how dogs respond to the shelter environment itself, particularly in terms of their ability to cope with acute stress.

Stress responses in dogs are highly variable, both in terms of environmental triggers and behavioral expression. Such behaviors reflect attempts to cope with acute stressors and may manifest as social withdrawal and behavioral inhibition or, conversely, as heightened activity, displacement behaviors, or aggression [14]. While these responses may appear concerning, they are often within the range of normal behaviors given the abrupt environmental change sheltered dogs face and may improve substantially once the dog is removed from the shelter setting. Nevertheless, certain stress-related behaviors, particularly those associated with arousal and excessive activity, such as jumping, circling, and elevated alertness, have been associated with decreased likelihood of adoption [15,16], complicating efforts to achieve both positive welfare and successful placement outcomes. As shelters work to promote welfare and place dogs as well-adjusted companions, the ability to rapidly identify individuals who are not coping effectively and to intervene early is critical.

Although several welfare assessment tools have been developed [17,18], few are designed to evaluate stress during the initial days following intake. This gap is significant, as behavioral deterioration within the first week occurs even in dogs that do not initially display overt signs of distress [19]. Therefore, timely and repeated assessment early in the shelter stay is essential. The objective of the present study was to evaluate a behavior assessment tool [LIFE (Living Interactions and Functionality Evaluation)] that is based on observation rather than provocation, with the aim of accurately characterizing coping ability in recently admitted shelter dogs and identifying those in need of early intervention.

## 2. Materials and Methods

### 2.1. Subjects and Housing

Subjects included all dogs aged ≥5 months who were housed at Gigi’s, a limited-admission, private dog shelter located in Canal Winchester, OH, USA, between November 2022 and February 2025. Dogs in this shelter primarily originate from one of six partner shelters in Ohio and are transferred via Ford Transit van, with transport times ranging from 45 min to 2.5 h.

In this shelter, housing consists of multiple indoor-only units, each containing 5–11 double-sided, double-doored, guillotine kennels with solid, 245 cm cinder block walls. Dogs are singly housed unless noted to be a “bonded pair”, in which case two dogs might share one double-sided kennel. One housing unit, reserved for nursing dams or shy/fearful dogs, has 5 rooms with floor-to-ceiling cinder block walls, a 2′ × 2′ window, and a glass-paned door facing an interior vestibule. Each housing unit has a walkway on either side of the kennel doors, with one exit leading directly outdoors and another leading to internal shelter hallways. The outdoor areas provide a mix of grass, concrete sidewalks, and asphalt parking lots, spanning approximately 6 acres of land.

Dogs are leashed, walked three times per day, allowed off-leash play time with humans and/or other dogs when deemed medically and behaviorally appropriate, and given enrichment, which could include food-stuffed puzzles, scented items, or human interaction a minimum of three times daily.

### 2.2. Behavior Assessment Tool

All dogs at this shelter are evaluated using the LIFE assessment tool (a draft is available upon request of the corresponding author). The assessment was designed to evaluate real-time coping ability across multiple contexts (“snapshots”) that are already standard interactions with shelter staff, including approach and interaction with other dogs (“dog interactions”), the kennel environment (“kennel”), leash walking (“leash”), and social interactions with humans (“human interactions”) in a living room-style, quiet room.

The “kennel” snapshot (S1A) records behaviors in response to the evaluator approaching the kennel, leashing the dog, and walking the dog out of the kennel. The “leash” snapshot (S1B) records behaviors observed walking from the kennel to the outside, walking around the campus for an elimination opportunity, and then walking into a quiet room. The original LIFE included both the “kennel” and “leash” behaviors in a single snapshot (S1). After noting that coping abilities varied greatly in these two scenarios, the shelter elected to score these interactions as two distinct snapshots with separate objective scores and subjective classifications.

The “human interactions” snapshot (S2) records behaviors observed when inside the quiet room while the evaluator interacts with the dog in various non-confrontational ways, such as speaking softly to them and offering pets, toys, and food treats. The dog is then allowed to settle during 10 min of quiet time, while the evaluator shifts focus off of the dog and onto a phone, tablet or book so that they are otherwise disengaged from the dog, aside from recording observed behaviors. As the goal is to capture behavior in naturally occurring interactions without adding provocation or stress beyond what is inherently present in the shelter environment, interactions between the evaluators and dogs are not scripted and, as such, may vary greatly. Evaluators adapt their own body language and behavior to each dog, minimizing directness and physical contact when the dog’s body language and behavior warrant distance.

The “dog interactions” snapshot (S3) attempts to introduce the dog to an unfamiliar dog, utilizing parallel walking at a distance of at least 25 feet, allowing for a gradual approach and then direct interaction on a leash, as long as neither dog is displaying fearful or aggressive body language. All evaluators are Fear Free Shelter^®^ Certified and have received training in defensive dog handling [20].

**Figure 1:**
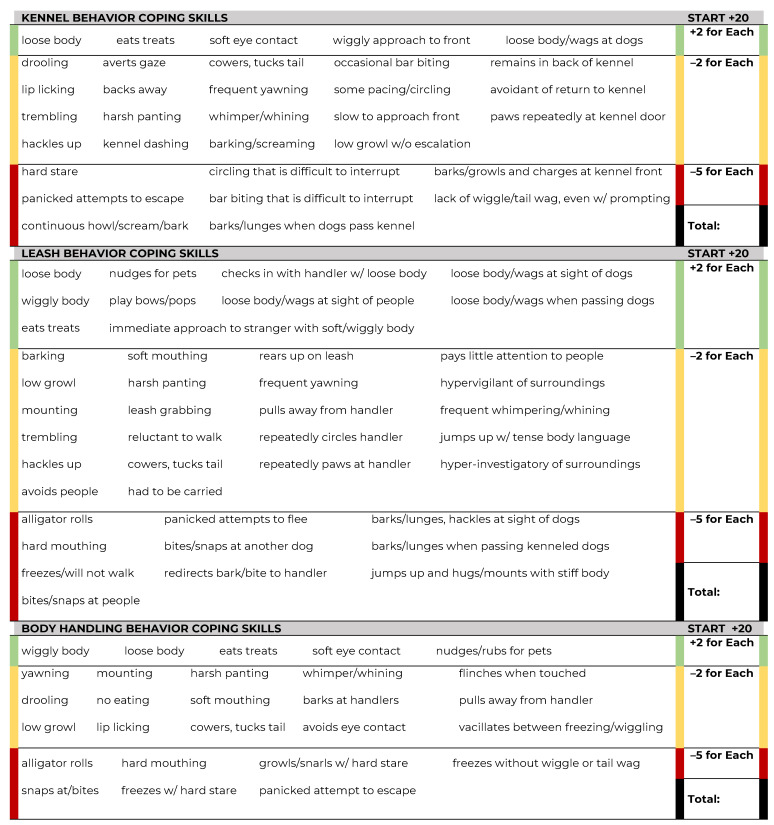
Sample scoring sheet for the LIFE tool. A numerical coping score is calculated based on observed behaviors. Behaviors associated with positive emotional states and effective coping contribute positive points and are designated as “green” behaviors. Behaviors indicative of negative emotional states and poor coping result in point deductions and are designated as either “yellow” or “red”, with “red” behaviors being those consistent with panic, aggression, and more dire coping.

For each snapshot, specific observable behaviors are assigned predetermined point values. Specific point values were established by a board-certified veterinary behaviorist based on repeated trials during initial development and pre-testing [21] during the timeframe between June 2020 and November 2022. Evaluators circle color-coded behaviors observed when engaging with the dogs in each of these snapshot interactions. Behaviors associated with positive emotional states [22] and effective coping contribute positive points and are designated as “green” behaviors [23,24]. Behaviors indicative of negative emotional states and poor coping [25,26] result in point deductions and are designated as either “yellow” or “red”, with “red” behaviors being those consistent with panic, aggression, and more dire coping [23,24]. An “objective” coping score is calculated for each snapshot, with possible scores ranging from 0 to 120, depending on the snapshot. To minimize the likelihood of negative scores and limit mathematical and recording errors, a baseline value of 25 was added to each section (Figure 1).

Evaluators also assign a “subjective” classification on a 4-point scale of “excellent”, “very good”, “adequate”, or “poor”, based on their overall perception of the dog’s coping ability in each context. Evaluations are conducted by trained staff members who work primarily in behavior-related roles but do not have advanced academic training or degrees in animal behavior (“non-experts”).

#### 2.2.1. Timing of Assessment

On the day of intake, it is standard practice in this shelter that all dogs receive a physical examination and preventative care, including vaccination, heartworm, flea, and tick prophylaxis, and treatment for endoparasites. Dogs then spend time with staff whose work is primarily focused on behavior within the next 24–48 h; these interactions form the basis of the LIFE assessment coding. Implemented in 2020, the assessment tool was designed to improve objective data collection and facilitate timely behavioral interventions. Dogs are typically evaluated with this tool between 1–2 days after intake by one of three behavior team members. Because it assesses coping in real time, evaluations may be repeated to track changes and responses to interventions. Dogs who are adopted and returned to the shelter after 30 days or longer follow the same intake process regarding medical and behavioral assessment.

#### 2.2.2. Validation Assessments

Validation is the process of assessing how well a test or measure performs by examining specific test qualities such as reliability and validity [27,28]. Reliability is a determination of the consistency of a measure over multiple measurements [28]. Inter-rater reliability (IRR) is particularly important when measuring behavior because behavioral observations usually require human judgment to identify, interpret, and record behaviors, making them susceptible to observer bias, differences in interpretation, and drift over time [29]. In addition, behavioral assessments used in shelters are implemented by multiple individuals, making high IRR an important test characteristic. High IRR indicates that observers are applying operational definitions consistently, increasing confidence that recorded differences reflect true behavioral variation rather than differences among observers. In order to evaluate IRR for the objective scoring of the LIFE assessment tool, 33 assessments of 30 dogs were conducted simultaneously by two “non-expert” trained evaluators from a pool of three trained staff members on S1 (kennel and leash behavior), S2 (human interactions), and S3 (dog interactions).

Validity is commonly thought of as the accuracy of the measure or, as described by American Educational Research Association et al. (2005), “the degree to which evidence and theory support the interpretations of test scores for proposed uses of tests” [28] (p. 11). Strong validity indicates that the measure accurately captures the construct it is intended to assess and that the resulting data meaningfully represent that construct. Establishing validity is essential because even highly reliable measures have limited scientific or practical value if they do not measure what they are intended to measure or do not support appropriate interpretations of the results.

Validity is commonly evaluated through multiple forms of evidence, including content validity, construct validity, and criterion-related validity, each of which provides different information about how well a measure represents the intended construct [27,28]. To establish the criterion validity of the LIFE assessment tool, a comparison of non-expert evaluator objective scores to expert subjective classifications was conducted while observing the assessments of 44 dogs. One of two expert evaluators with advanced degrees/certification in animal behavior provided subjective classifications for Snapshot 1 in total (kennel and leash behavior), not by sub-part (S1A/B), as the previous version of the LIFE tool treated this snapshot as a single assessment at that time.

In addition, one of three trained, non-expert evaluators provided objective scores and subjective classifications for 852 assessments of 832 dogs. Twenty assessments included dogs that had been evaluated with the LIFE tool at two separate timepoints, due to being returned to the shelter after being previously adopted. These single-evaluator assessments were treated as evidence of convergent validity between objective and subjective ratings.

### 2.3. Statistical Analysis

IBM SPSS v29 was used for analysis of all available LIFE data. Frequencies were calculated for all categorical data such as subjective classifications. Descriptive statistics were calculated for all numeric data including objective scores.

IRR was calculated for the objective scores using interclass correlation (ICC) in a two-way mixed, absolute, average-measures model [29]. An ICC result of 0.80 or higher was considered excellent, following the guidance of Cicchetti, 1994 [30].

Descriptive statistics for evaluator objective scores were calculated for each subjective classification (excellent, very good, adequate, and poor) as assessed by evaluators and expert evaluators and examined for significant trends in scores by assessment grouping using Jonckheere–Terpstra tests, a nonparametric test based on ranks that can identify a statistically significant monotonic trend between an ordinal independent variable and a continuous or ordinal dependent variable [31].

*p*-values less than 0.05 were determined to be statistically significant.

## 3. Results

### 3.1. Demographics

Demographic characteristics are presented for the 862 dogs involved in the 885 independently completed evaluations (33 objective reliability evaluations on 30 dogs; 852 standard evaluations on 832 dogs), although some dogs were missing demographic data. Of these dogs, 855 had valid sex and alter status data, with most being intact at the time of the assessment: 348 (40.7%) were intact females and 357 (41.8%) were intact males, with just 75 (8.8%) spayed females and 75 (8.8%) neutered males. The mean age of the dogs at the time of evaluation was 31.0 months (SD = 25.4, Median = 24.0, min = 5.0, max = 168.0, n = 840). Mean weight was 20.1 kg (SD = 8.3, Median = 20.0, min = 2.7, max = 67.7, n = 853). Fifty-five breeds including mixed breeds were recorded for 862 dogs, with the most frequent being mixed breed (n = 622, 72.2%), followed by beagle (n = 31, 3.6%) and German shepherd dog (n = 22, 2.6%). Of the mixed-breed dogs, 278 (32.3% of all dogs) were classified as large (over 20 kg), 271 (31.4% of all dogs) as medium (11–20 kg), and 73 (8.5% of all dogs) as small (under 11 kg). Breed was assigned based on visual appearance per standard practice at this shelter. Since visual assignment of breed status is prone to inaccuracy [32], dogs were classified as mixed breed unless they had an unequivocal phenotype consistent with a purebred dog.

### 3.2. Reliability

Two non-expert evaluators both conducted 33 independent evaluations on 30 dogs for S1, S2, and S3. The ICC was excellent [30], ranging from 0.822 to 0.955 across the three snapshots, with a mean ICC of 0.890.

### 3.3. Validity

#### 3.3.1. Evaluator Objective Scores and Expert Subjective Assessment

Expert evaluators assessed 44 dogs on S1, S2, and S3 using the subjective classifications; these subjective classifications served as a form of criterion validity for the evaluators’ objective scores of these same dogs. Table 1 presents descriptive statistics for evaluator objective scores based on expert subjective classifications. Note that subjective classifications for S1 are in total, not by sub-part. Jonckheere–Terpstra tests determined that there were statistically significant increasing monotonic trends in scores for all snapshots, *p* < 0.001, meaning that as expert subjective assessments improved, evaluator scores increased (Snapshot 1: J-T = 407.5, *p* = 0.001; Snapshot 2: J-T = 416.0, *p* < 0.001; Snapshot 3: J-T = 357.5, *p* < 0.001).

**Figure 2:**
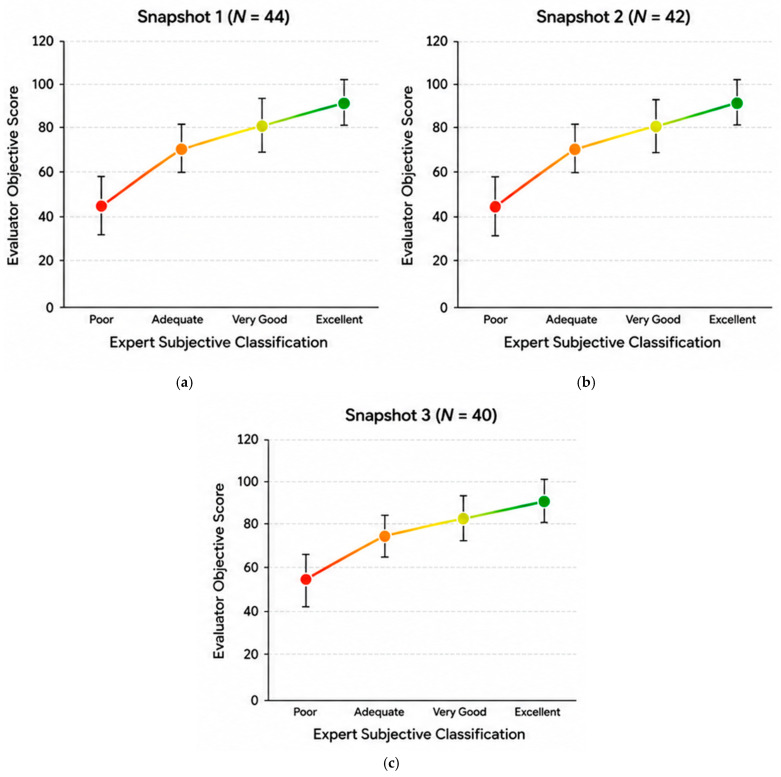
(**a**–**c**)**.** Mean evaluator objective scores based on expert subjective classifications of “poor”, “adequate”, “very good”, or “excellent” with error bars representing one standard deviation from the mean for S1 (**a**), S2 (**b**), and S3 (**c**), with corresponding behavior color coding in the “rainbow” line. A numerical coping score is calculated based on observed behaviors. Behaviors associated with positive emotional states and effective coping contribute positive points and are designated as “green” behaviors. Behaviors indicative of negative emotional states and poor coping result in point deductions and are designated as either “yellow” or “red”, with “red” behaviors being those consistent with panic, aggression, and more dire coping. N refers to the number of dogs.

As subjective scores improved, objective scores showed corresponding numerical increases, aligning with the red, yellow, and green designations for negative or positive emotional states (Figure 2a–c).

#### 3.3.2. Evaluator Objective Scores and Evaluator Subjective Classifications

Convergent validity of the objective scores was investigated by comparing evaluator objective scores to evaluator subjective classifications of these same dogs. Table 2 presents descriptive statistics for evaluator objective scores based on evaluator subjective classifications. Data from 228 assessments were available for analysis after the separation of S1 into two separate parts, reported as S1A and S1B. One hundred twelve (49.0%) of the evaluations performed after this separation had dogs with subjective classifications for S1A that differed from their subjective classification for S1B, justifying treating these parts of S1 separately. A total of 822 assessments had valid data for S2 and 815 had valid data for S3.

Jonckheere–Terpstra tests determined that there were statistically significant increasing monotonic trends in scores for all snapshots, *p* < 0.05, meaning that as subjective assessments improved, scores increased (Snapshot 1A Kennel Behavior: J-T = 14549.5, *p* < 0.001; Snapshot 1B Leash Behavior: J-T = 14,366.0, *p* < 0.001; Snapshot 2: J-T = 211,588.5, *p* < 0.001; Snapshot 3: J-T = 188,572.0, *p* < 0.001).

**Figure 3:**
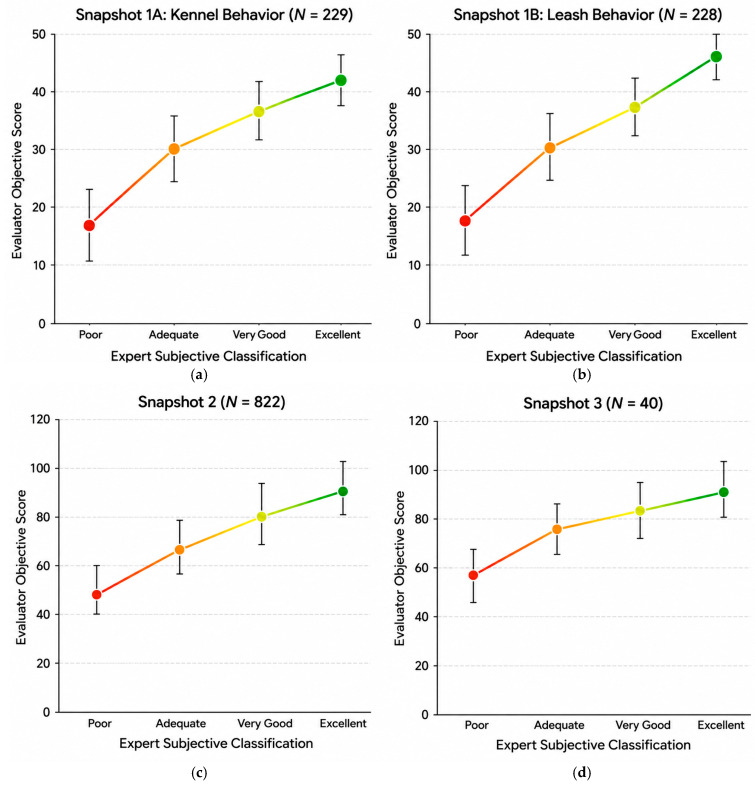
(**a**–**d**). Mean evaluator objective scores based on evaluator subjective classifications of “poor”, “adequate”, “very good”, or “excellent” with error bars representing one standard deviation from the mean for S1A (**a**), S1B (**b**), S2 (**c**) and S3 (**d**), and corresponding behavior color shown by the diagonal “rainbow” line. A numerical coping score is calculated based on observed behaviors. Behaviors associated with positive emotional states and effective coping contribute positive points and are designated as “green” behaviors. Behaviors indicative of negative emotional states and poor coping result in point deductions and are designated as either “yellow” or “red”, with “red” behaviors being those consistent with panic, aggression, and more dire coping. N refers to the number of assessments.

As subjective scores improved, objective scores showed corresponding numerical increases, aligning with the red, yellow, and green designations for negative or positive emotional states (Figure 3a–d).

## 4. Discussion

The findings of this study provide strong support for the LIFE tool as a valid and reliable method of assessing how well dogs are coping within the shelter environment. Objective scores generated by the tool demonstrated clear, statistically significant alignment with expert subjective assessments across multiple contexts within the sheltered dog’s experience. These results indicate that the numerical scoring system meaningfully reflects expert interpretation of canine coping, offering a standardized approach to in-shelter behavior assessment.

By reducing reliance on purely subjective interpretation, the LIFE tool increases consistency across evaluators and improves the ability to track changes in behavior and coping abilities over time. High inter-rater reliability further supports that trained non-expert staff can apply the tool consistently, reinforcing its feasibility for widespread implementation in shelter settings without requiring advanced behavioral credentials.

The splitting of S1 into two distinct kennel (S1A) and leash/outdoor (S1B) assessments emerged as a particularly meaningful refinement of the tool, with nearly half of the dogs receiving different classifications across these two contexts. Some dogs appear relatively comfortable within the kennel but demonstrate signs of stress when handled on leash or exposed to outdoor environments, while others cope well outside the kennel yet struggle with confinement or indoor stimuli. Evaluating these contexts separately allows for more precise identification of stressors and supports the development of more targeted interventions. Given that both kennel confinement and leash walking are near-universal aspects of shelter life, this distinction enhances both the sensitivity and practical utility of the assessment.

A key strength of the LIFE assessment lies in its foundation in naturally occurring interactions. Unlike traditional behavioral evaluations that rely on standardized, and often provocative, test scenarios [3,5,8,9], the LIFE tool captures behavior during routine shelter activities that dogs already experience. As such, the tool does not require additional time-intensive procedures or the introduction of artificial stressors that may produce unsafe or misleading results. Instead, implementation primarily requires training evaluators to reliably recognize and score defined behaviors, allowing shelters to integrate the assessment into existing workflows with minimal disruption.

This observational approach is particularly important given longstanding concerns about the validity and predictive limitations of many commonly used traditional shelter behavior tests [6,7]. By focusing on how dogs respond to real-world shelter conditions, the LIFE tool aligns more closely with its intended purpose: assessing in-shelter coping rather than predicting behavior in future environments. Importantly, this tool is not intended to serve as the sole adoptability assessment nor to predict post-adoption behavior. A substantial body of evidence indicates that certain behaviors displayed in the shelter are not strong predictors of behavior in the home [6,7,8]. Instead, the LIFE tool should be understood as a welfare-focused instrument designed to identify dogs experiencing difficulty coping with the shelter environment and to guide supportive interventions. As suggested by Clay et al. [1], behavior assessments, including information gathered from the LIFE tool, are part of a suite of measures to better understand a dog’s characteristics and adoptability, along with previous owner information as well as foster, staff, and volunteer observations.

An additional implication of this work is early identification and intervention for dogs who are not coping well. Since the purpose is not to determine personality or in-home temperament, it can be utilized at any point in a sheltered dog’s journey, including both acute and chronic stress phases. Hennessey et al. described elevated stress hormone levels during the initial 3 days after intake into a shelter, with moderate levels persisting for an additional 6 days [33]. Dogs experiencing unmanaged or escalating stress may exhibit behaviors that obscure their typical temperament [25]. Conversely, when stress is effectively mitigated, dogs could be more likely to display behaviors that are more representative of their baseline disposition. By facilitating earlier recognition of coping challenges, the LIFE tool may help shelters implement timely interventions that not only improve welfare but also allow dogs to more accurately express their behavioral tendencies.

Several limitations should be considered when interpreting the findings of this study. Although the LIFE tool was designed for use by non-expert staff, the evaluators in this study were trained individuals who worked primarily in behavior-focused roles. As a result, they may have been more adept at recognizing subtle behavioral indicators than less experienced shelter personnel. In practice, shelters adopting this tool should require additional training in animal behavior and body language interpretation, not only for their safety, but also to ensure reliable identification and scoring of specific behaviors. This need for training is especially relevant in cases involving socially withdrawn dogs. Behaviors such as freezing, immobility, or avoidance during human interaction may be misinterpreted as relaxation or calmness without sufficient experience or guidance, potentially leading to inaccurate scoring and misclassification of coping ability.

While the objective scoring system demonstrated strong alignment with subjective classifications, a slight numerical overlap was observed between categories. Consequently, reliance on objective scores alone may result in misclassification, particularly for dogs whose scores fall near category thresholds. Rather than viewing these dogs as belonging exclusively to one category or another, we propose interpreting coping as a continuum. Building on the familiar “green–yellow–red” color framework commonly used to describe canine emotional states [23,24], these overlapping ranges support a more nuanced “coping rainbow,” where dogs with scores near category boundaries may be better represented by transitional colors (e.g., light green or orange) that acknowledge characteristics of both adjacent categories. This approach better reflects the biological reality that coping exists along a spectrum. Objective scores should therefore be interpreted in conjunction with contextual information and behavioral nuance, requiring a degree of informed subjectivity to ensure accurate assessment.

The wider interquartile ranges observed for Snapshot 2 (human interactions in a quiet room) likely reflect both meaningful behavioral heterogeneity and the intentionally flexible nature of this assessment. Compared with the other snapshots, Snapshot 2 provides a longer observation period during which dogs have opportunities to display a broader repertoire of behaviors, including affiliative interactions, play, food engagement, exploration, and passive settling. Consequently, there are more opportunities for both point additions and deductions, resulting in greater score variability. Additionally, because evaluator–dog interactions are intentionally unscripted and adapted to each dog’s responses, some variation in evaluator behavior is expected and likely contributes to the broader score distribution. These findings further reinforce that objective scores should be interpreted within the context of the individual dog’s overall behavioral presentation rather than as rigid categorical cutoffs. Ideally, the LIFE tool should be used as a dynamic measure, with repeated assessments to monitor baseline coping and response to interventions, and never as a one-time determinant or predictor of outcome.

Future directions should focus on evaluating the generalizability of the LIFE tool across a wider range of shelter environments, including open-admission facilities and shelters with varying resource levels. Multi-site trials will be important to confirm robustness across different populations, operational contexts, and personnel. Standardization of the tool’s terminology and a defined ethogram will be important as multiorganizational use expands.

Additionally, the development of a companion intervention guide represents a critical next step. Such a resource would link specific scoring patterns to evidence-based strategies for reducing stress, thereby enhancing the tool’s practical impact and supporting shelters in translating assessment outcomes into meaningful welfare improvements.

## 5. Conclusions

The LIFE assessment tool provides a valid and practical framework for evaluating how well dogs are coping in shelter environments. Its numerical scoring system aligns with expert behavioral assessments while offering a standardized approach that can be consistently applied by trained, non-expert staff.

By focusing on naturally occurring interactions rather than provocative testing, the tool captures relevant behavior without increasing risk or resource demands. Its ability to distinguish coping across different contexts, particularly between kennel and leash/outdoor environments, enables more precise identification of stressors and supports targeted intervention strategies. With further validation and the development of supporting intervention resources, the LIFE tool has strong potential to serve as a widely applicable and impactful approach to advancing canine welfare in shelters.

## Figures and Tables

**Table 1:** Descriptive Statistics of Evaluator Objective Scores Based on Expert Subjective Classifications.

Evaluator Objective Score	Expert Subjective Classifications				Total
	Poor	Adequate	Very Good	Excellent	
Snapshot 1 **					
Mean	--	67.1	76.5	86.5	72.3
SD	--	11.5	6.7	3.5	10.8
Median	--	69.5	75.0	86.5	73.0
Min	--	40.0	63.0	84.0	40.0
Max	--	83.0	89.0	89.0	89.0
IQR	--	19.0	11.5	5.0	14.0
N	0	22	20	2	44
Snapshot 2 **					
Mean	44.0	69.9	80.5	88.5	74.9
SD	12.7	8.8	10.5	6.4	13.1
Median	44.0	71.0	79.0	88.5	75.5
Min	35.0	58.0	65.0	84.0	35.0
Max	53.0	82.0	99.0	93.0	99.0
IQR	18.0	16.0	18.0	9.0	16.0
N	2	17	21	2	42
Snapshot 3 **					
Mean	58.5	74.0	81.5	90.0	76.3
SD	3.5	8.2	6.7	5.7	9.6
Median	58.5	74.0	82.0	90.0	77.0
Min	56.0	58.0	71.0	86.0	56.0
Max	61.0	90.0	90.0	94.0	94.0
IQR	5.0	10.0	10.0	8.0	11.0
N	2	24	12	2	40

Note ** *p* < 0.01. N refers to the number of dogs.

**Table 2:** Descriptive Statistics of Evaluator Objective Scores Based on Evaluator Subjective Classifications.

Evaluator Objective Scores	Evaluator Subjective Classifications				Total
	Poor	Adequate	Very Good	Excellent	
Snapshot 1A Kennel Behavior **					
Mean	18.7	30.6	37.4	39.4	34.0
SD	6.9	6.2	2.6	1.1	7.4
Median	19.0	31.5	37.0	40.0	37.0
Min	6.0	15.0	29.0	37.0	6.0
Max	35.0	40.0	45.0	40.0	45.0
IQR	9.0	8.5	3.0	0.0	8.0
*N*	23	60	118	28	229
Snapshot 1A Leash Behavior **					
Mean	20.0	34.9	42.1	47.0	37.1
SD	7.0	7.1	4.9	3.0	9.2
Median	21.0	35.0	43.0	46.0	40.0
Min	7.0	14.0	26.0	43.0	7.0
Max	36.0	48.0	52.0	52.0	52.0
IQR	9.0	11.0	5.0	3.0	13.0
*N*	23	101	87	17	228
Snapshot 2 ** Human interactions					
Mean	48.8	65.5	78.7	89.4	73.8
SD	9.7	9.2	8.4	6.4	14.2
Median	48.0	66.0	79.5	89.5	75.0
Min	21.0	29.0	54.0	71.0	21.0
Max	72.0	85.0	103.0	111.0	111.0
IQR	13.0	12.5	10.0	8.0	20.0
*N*	62	300	292	168	822
Snapshot 3 ** Dog interactions					
Mean	53.4	69.7	79.1	86.1	75.5
SD	12.7	8.3	7.6	6.7	11.7
Median	56.0	70.0	80.0	86.0	76.0
Min	26.0	48.0	56.0	66.0	26.0
Max	78.0	88.0	100.0	98.0	100.0
IQR	17.0	14.0	10.0	10.0	16.0
N	56	267	339	153	815

Note ** *p* < 0.01. N refers to the number of assessments.

## Data Availability

The data presented in this study are available on request from the corresponding author due to organizational privacy restrictions.

## Abbreviations

The following abbreviations are used in this manuscript:

LIFE	Living Interactions Functionality Evaluation
S1	Snapshot 1: Kennel and Leash Behavior (Combined)
S1A	Snapshot 1A: Kennel Behavior
S1B	Snapshot 1B: Leash Behavior
S2	Snapshot 2: Human Interactions
S3	Snapshot 3: Dog Interactions
ICC	Interclass Correlation
IRR	Inter-Rater Reliability
